# Preparation, and Assessment of Antidermatophyte Activity of Miconazole–Urea Water-Soluble Film

**DOI:** 10.3389/fmicb.2020.00385

**Published:** 2020-04-03

**Authors:** Omar Y. Mady, Lamiaa A. Al-Madboly, Ahmed A. Donia

**Affiliations:** ^1^Department of Pharmaceutical Technology, Faculty of Pharmacy, Tanta University, Tanta, Egypt; ^2^Department of Pharmaceutical Microbiology, Faculty of Pharmacy, Tanta University, Tanta, Egypt; ^3^Department of Pharmaceutical Technology, Menoufia University, Shebeen El-Kom, Egypt

**Keywords:** medicated film, dermatophytosis, guinea pig model, miconazole/urea, MIC/MBC

## Abstract

Cutaneous mycoses, particularly tinea pedis caused by *Trichophyton rubrum*, are commonly known infections in humans. They are still considered as a major public health problem worldwide affecting the quality of life due to prolonged period of treatment and development of drug resistance, which leads to recurrence of infections. The objective of our study was to assess the effectiveness of miconazole in the presence and absence of urea, as a penetration enhancer, against *T. rubrum* and to formulate both of them in a water-soluble film to be applied topically for the purpose of treating tinea pedis caused by this fungus. Drug combination revealed synergism where miconazole minimum inhibitory concentration (MIC) and minimum fungicidal concentration (MFC) (0.5 and 1 mg/L) were considerably declined to 0.001 and 0.004 mg/L, respectively, when combined with 20% urea. This enhanced drug interaction activity against the test strain was explained by the alterations raised on the morphology and ultrastructures observed microscopically. Minimal fungicidal dose of miconazole/urea combination displayed plasmolysis and shrink cytoplasm; however, necrotic cells with punctured walls and degraded cytoplasmic content were observed at high fungicidal dose. Water-soluble films, prepared using increasing values of miconazole MFC and urea, were transparent, smooth, uniform, and flexible. Their physicochemical characters showed homogeneity in weight, thickness, drug content, and folding endurances with normal surface pH values, indicating the reproducibility of the preparation method. The novel simulation model for the film mechanism of action supported the idea and the suggested application method of the new dosage form. Evaluation of these films was carried *in vitro* using disk diffusion assay as well as *in vivo* using guinea pig dermatophytosis model. The *in vitro* assessment revealed an increase in the inhibition zone diameters in a concentration-dependent manner upon using 10 or 20% of urea combined with miconazole. *In vivo* test showed that combination of 0.004 mg/L miconazole with 20% urea (M + U20) showed the highest efficacy percentage (95.83%), which was statistically superior to the infected untreated control (*p* < 0.001) in fungal burden reduction as well as improvement in clinical scores (*p* < 0.001). This work supports the hypothesis and suggests a new promising dosage form for the treatment of *T. rubrum* infections.

## Introduction

*Trichophyton rubrum*, the most prevalent dermatophyte, is attributed to 80% of dermatomycoses affecting keratinized tissues of humans, and so they are anthropophilic. It uses keratin as a nutrient during scalp, skin, and nail infections (Komoto et al., 2015). In cutaneous lesions, the pathogen adheres and may penetrate the host tissue to scavenge nutrient macromolecules as carbon, nitrogen, phosphorus, and sulfur sources. Therefore, deeper infections may occur due to overcoming the host defense mechanisms. This is most commonly detected among immune-deficient individuals, particularly those treated with immunosuppressive agents or long-term use of corticosteroids (Peres et al., 2010). Symptoms range from mild to severe according to the host’s immunological condition. Typical lesions of the infected skin (interdigital tinea pedis or athlete’s foot) are macerated, erythematous, pruritic, peeling, and burning, which may be due to the direct effect of the fungus itself or its metabolic end products (Degreef, 2008).

The antifungals imidazole (ketoconazole, miconazole) and triazole (fluconazole, itraconazole) have been used to treat dermatophytosis for many decades. Azole antifungals inhibit sterol 14a-demethylase in the ergosterol biosynthesis pathway (Cardoso et al., 2016). However, infections caused by *T. rubrum* are difficult to treat in spite of a reasonable number of antifungal drugs available for clinical use, but many of them induce adverse effects. In addition, therapeutic effectiveness may also be reduced because infections generally are associated with high recurrence (Cardoso et al., 2016; Lima et al., 2017). Specifically, *T. rubrum* has developed resistance to the principal antifungal agents, especially to azole drugs such as ketoconazole and fluconazole. The principal molecular resistance mechanisms involve changes in the drugs’ target enzymes, high expression of efflux pumps, and changes in permeability or uptake of the drug. The application of local antifungal has a low chance to penetrate the thick wet skin and hence develop resistance to the drug (Lima et al., 2017).

Several studies have investigated the effect of different drugs, either synthetic or natural products, on *T. rubrum*, but there are no reports about the use of miconazole/urea combination against the test fungus (Smijs et al., 2008; Pereira et al., 2011; Kingsbury et al., 2012; Aala et al., 2014; Song et al., 2018). Interestingly, Mady et al. (2018) developed novel buccal muco-adhesive patches against *Candida albicans*, which have the ability to attach to the buccal cavity, exerting a local effect on the infected site. In addition, the authors used urea in the prepared muco-adhesive patches, for the first time, which is a well-known penetration enhancer in the pharmaceutical technology to overcome the resistance of the pathogen. The use of this combination led to a dramatic decrease in the minimum inhibitory concentration (MIC) of the antifungal drug due to the dual effect of the later drug and the penetration enhancer, leading to a decrease in the adverse effects of the antifungal agent. This conclusion encouraged the authors to use the same combination for treatment of tinea pedis but in an innovative pharmaceutical dosage form (water-soluble film) to be applied as a stripe (thin film), which can be located between the infected foot fingers.

Treatment of tinea pedis faces two problems. First, the infected skin is characterized by being extremely thick. This is attributed to the keratinization, an important defense mechanism of the host toward the fungus, by keratinocytes responsible for the stratum corneum renewal process. Second, the causative agent may invade the deeper layers in immunocompromised patients (Peres et al., 2010). Therefore, it is essential to evaluate the antifungal activity *in vivo* using appropriate animal model. Guinea pigs were reported in the literature to be suitable as a dermatophytosis model because they are susceptible to fungal infection as human. In addition, they have large body surface, giving a sufficient area to do tests and hence determine the clinical as well as the mycological efficacies. Garvey et al. (2015) and Long et al. (2016) used a guinea pig model to assess the efficacies of various antifungals for the treatment of dermatophytosis. Accordingly, the aim of this work is to evaluate the combined effect of miconazole as an antifungal agent as well as urea as a penetration enhancer against *T. rubrum in vitro* and *in vivo*. This combined therapy should be prepared in a special pharmaceutical dosage form to be easily applied between the infected fingers, adsorb the watery secretion from the skin for its solubilization, and then deliver its contents for local pharmacological effect.

## Materials and Methods

### Materials

Miconazole base and carboxymethyl cellulose (CMC) were obtained as a gift sample from Sigma Pharmaceutical Company, Quesna, Egypt. Propylene glycol was purchased from BDH Chemical Ltd. (Poole, England).

#### Test Microorganisms

*Trichophyton rubrum* (ATCC 28188) and *Trichophyton mentagrophytes* (ATCC 24953), the test strains and the most common causes of human onychomycosis, were obtained from the Department of Pharmaceutical Microbiology, Faculty of Pharmacy, Tanta University, Tanta, Egypt.

A guinea pig model was used to assess the efficacies of various water-soluble films formulated for the treatment of dermatophytosis. We used *T. mentagrophytes* (ATCC 24953) to infect guinea pigs, since this is a zoophilic fungus and it is capable to infect the animals’ skin, leading to an inflammatory reaction in the skin as well as hair root invasion. In the contrary, *T. rubrum* is an anthropophilic fungus and fails to cause infection. Furthermore, *T. mentagrophytes* is a fungus responsible for human cutaneous infections so that it is clinically relevant.

#### Laboratory Animals

Male albino guinea pigs aged 12–15 weeks with a body weight of 450–500 g were purchased from the Laboratory Animal Center of Helwan University, Helwan, Egypt. They were housed in the animal house of the Faculty of Pharmacy, Tanta University, Tanta, Egypt. The protocol procedures followed the guidelines of the Animal Welfare Act, the Guide for the Care and Use of Laboratory Animals, and the Office of Laboratory Welfare (2011). The animal infection protocol was reviewed and approved by Animal Care and Use Committee at the Faculty of Pharmacy, Tanta University, Tanta, Egypt with approval number (REC-TP/M0003).

The environmental controls of the animal room were adjusted. Temperature was set at 16–22°C, and the relative humidity was maintained at 30–70%. In addition, a 12-h light and 12-h dark cycle was also adjusted. Experimental animals were subjected to an acclimation period for 5 days before use with free access to food and water (in a bottle with nozzle) and housed in a chew-proof wire cage with plastic base for easy cleaning (30 L × 18 W × 16.5 H). In addition, they were clinically observed daily with measurement of body weight. All experimental procedures were carried out while the animals were generally anesthetized, and all efforts were done to minimize animal suffering.

### Methods

#### Antifungal Susceptibility Testing of Miconazole Against *T. rubrum* and *T. mentagrophytes*

The MIC of miconazole was carried out, in the absence and presence of urea, using broth microdilution method according to the standard procedure described in M38-A2, which is a document prepared by the Clinical and Laboratory Standards Institute (CLSI, 2008), with some modifications. Sterile microtiter plates with 96 round-bottom wells were utilized. Roswell Park Memorial Institute (RPMI) 1640 with L-glutamine was used as a suitable medium for the test. It was supplemented with 2% glucose and buffered with 0.165 M morpholinepropanesulfonic acid (MOPS) to pH 7.0. A stock solution of miconazole dissolved in dimethylsulfoxide (DMSO) was prepared, and then twofold serial dilutions, with double-strength RPMI with 1% DMSO, were carried out to give a final concentration range of 64–0.03125 mg/L. Similarly, urea was diluted to give a final concentration range of 40–0.039%. About 20-μL aliquots of each drug concentration were added to the wells of the microtiter plate. Testing of the addition of 10, 20, or 40% urea to miconazole was carried out in a similar way to evaluate miconazole/urea combinations where 20-fold concentrations of each drug were serially diluted twofold, and 10-μL aliquots were added to the wells of the microtiter plates using the checkerboard method. Microconidia of *Trichophyton* spp. were prepared in a concentration of 1.1 × 10^4^ microconidia/mL by washing them off Sabouraud dextrose agar overnight culture using sterile distilled water. The suspension was filtered through 8-μm Whatman filter, and viable count was performed for purposes of quality control to confirm the proper well density. About 180 μL of the fungal cells was transferred to the 20-μL drug dilutions in the wells. Positive control wells contained 100 μL of RPMI medium mixed with 100 μL of inoculum, while negative controls were composed of 200 μL of RPMI only, and both were included in all experiments. In addition, wells for the drug solvent (RPMI with 1% DMSO) were also considered. Sterility control wells contained 100 μL of sterile distilled water. All plates were sealed with parafilm and then incubated for 5 days at 30°C (skin temperature). After incubation, the optical density (OD) was measured at a wavelength of 600 nm using a Tecan^TM^ Sunrise plate reader (Austria). Blank value was subtracted from the readings of the other wells. The MIC_80_ was defined as the lowest concentration of the drug that led to at least 80% reduced growth compared with untreated control.

To calculate the minimum fungicidal concentration (MFC), the whole content of each well displaying no visible growth was transferred to Sabouraud dextrose agar. It is defined as the lowest concentration of the drug resulting in 99% reduction in the viable colony-forming unit (CFU) from the inoculum. All experiments including MIC and MFC were carried out in triplicate.

The fractional inhibitory concentration (FIC) was determined to quantify the drug interaction, and it was calculated as follows: FIC = (MIC_80_ of drug A in combination/MIC_80_ of drug A alone) + (MIC_80_ of drug B in combination/MIC_80_ of drug B alone). Data were interpreted according to the American Society for Microbiology (ASM) guidelines: synergism if FIC is ≤ 0.5, indifferent if > 0.5 and ≤ 4, and antagonism if FIC is > 4 (Kingsbury et al., 2012).

### Effects on Morphology and Ultrastructures

#### Inverted Phase Contrast Microscopy

The morphological changes of *T. rubrum* following the drug or drug combination treatment were investigated in a 96-well microtiter plate with flat bottom. About 1.1 × 10^4^ microconidia/mL of the test fungus were prepared and treated with 0.25, 0.5, and 1 mg/mL of miconazole, 10 or 20% urea alone or combination of miconazole (0.001 or 0.004 mg/mL) with 20% urea in RPMI medium without phenol red and incubated at 30°C for 3 days before the examination under a phase-contrast inverted microscope (×400). DMSO-treated *T. rubrum* was used as a negative control. Alterations detected in the test fungal structures were recorded, photographed, and compared with the normal growth in the negative control group (Pereira et al., 2011; Song et al., 2018).

#### Transmission Electron Microscopy

Test specimens were primarily prepared for electron microscopy at the Microbiology Laboratory, Department of Pharmaceutical Microbiology, Faculty of Pharmacy, Tanta University, Egypt. *T. rubrum* was treated with 1/2 MIC (0.25 mg/L) of miconazole, 20% urea, or a combination of both 20% urea and 0.001, 0.002, or 0.003 mg/L of miconazole in RPMI medium for 14 days at 30°C. Fungal cells were harvested by precipitation in the microfuge at 4000 r/min for 15 min, and then, the pellet was washed twice with phosphate-buffered saline (PBS, pH 7). Next, samples were dispersed in the fixative solution and sent to the Electron Microscope Unit, Faculty of Medicine, Tanta University for transmission electron microscopy (TEM).

### Preparation of Water-Soluble Film

Preparation of soluble films was dependent on the well-known solvent casting method (Mahajan et al., 2011). Hot water was used to dissolve CMC polymer where a clear solution was produced. The quantity of either urea, miconazole, or combination of them was dissolved in the prepared polymer solution. Then, while stirring the mixture, the required volume of propylene glycol was added as a plasticizer. The prepared solution was casted in a Petri dish (78.6 cm^2^) and dried in an oven at 40°C. Following complete dryness, circular patches with 4-cm^2^ surface area were cut to evaluate the physicochemical properties as well as the microbiological studies.

### Evaluation of the Prepared Film

#### Testing the Physical Characters

The physical characters of the prepared films were studied according to the reported standard methods (Semalty et al., 2008; Khairnar et al., 2009; Giradkar et al., 2010). The uniformity of the prepared film weight was controlled gravimetrically. Three individual films with a surface area of 4 cm^2^ were weighed, and the results were analyzed for mean and standard deviation (SD; Semalty et al., 2008). The thickness homogeneity of the prepared films was also investigated, where three samples of them were measured using a Vernier caliper, and the results were presented as mean and SD (Giradkar et al., 2010). In addition, the folding endurance of the prepared films was also controlled by folding the films until breaking or up to 200 times (Khairnar et al., 2009).

#### Instrumental Analysis Tests

##### Surface pH

The surface pH of the prepared film was investigated using a pH meter. According to Khairnar et al. (2009), the prepared film should be immersed first in 5 mL distilled water for 5 min. Then, the surface pH was measured using a combined glass electrode at ambient temperature. The experiment was performed in triplicate.

#### Drug Content of the Prepared Films

A calibration curve for the drug was constructed and validated. The effect of the film constituents on the drug absorbance was also tested. Then, an area of the medicated film (4 cm^2^) was dissolved in 10 mL phosphate buffer, pH 6.8, and the resultant solution was subjected to drug content determination using a UV spectrophotometer (Thermo Fisher Scientific, Madison, WI, United States). The absorbance of the drug was determined at λ_max_ of 272 nm as reported by Hiremath et al. (2009). The actual drug content (ADC) was expressed in mg/4 cm^2^.

#### Local Film Solubility Model

The following model represented the expected mechanism of action of the topically applied film. A filter paper was immersed in distilled water for 5 min. After that, the filter paper was carefully removed and shaken to discard the excess water, then weighed. A known weight of the film was placed over the wet filter paper. At a time interval, the film was carefully removed and weighed. This procedure was repeated until the medicated soluble film lost the possibility to pick up for weighing. The weight of the test film was calculated at different time intervals according to the following equation:

Testfilmweightpercent=(W-tW)0/W0

where *W*_*t*_ is the film weight at time *t* and *W*_0_ is the film weight at time 0. The test film solubility curve was drawn as film weight against time. Therefore, the test film solubility curve represented the absorption of secretions from moist infected area, which was considered the first step in the treatment, resulting in dryness of the infected area and release of the active ingredients.

### *In vitro* Microbiological Assessment of the Drug-Containing Films Against Test Strains

Kirby Bauer disk diffusion method was used to evaluate the antifungal activity of drug-containing films with some modifications (Bauer et al., 1966). Briefly, 0.6 cm circular films containing 0.004 mg/L miconazole (M), 10% urea (U10), 20% urea (U20), 40% urea (U40), a combination of both together (M + U10, M + U20, or M + U40), and placebo film (P) were prepared. The test films were laid over the surface of Sabouraud agar seeded with 1 mL of 0.5 McFarland *T. rubrum* or *T. mentagrophytes* microconidia in 0.85% *w*/*v* sterile saline and adjusted the final concentration to be 2.5 × 10^3^ to 5 × 10^3^ CFU/mL. After incubating the plates at room temperature for 14 days, they were investigated for the presence of inhibition zones, which were measured and expressed in millimeters. All the experiments were performed in triplicates, and the diameter means were calculated as well as the SD.

#### Guinea Pig Dermatophytosis Model

*Trichophyton mentagrophytes* ATCC 24953 strain was subcultured on Sabouraud agar and incubated at 30°C for 5 days. A suspension of 1 × 10^7^ conidia/100 μL was prepared using sterile saline (0.85% NaCl). Animals were subjected to general anesthesia intramuscularly using 0.2 mL cocktail of ketamine, xylazine, and acepromazine (3:3:1, by volume). Using an electric razor, hair was clipped on the left side of the animals’ back. Closer shave was performed using a disposable razor. A stencil and marking pen were used to draw a square (2.5 cm × 2.5 cm), and the skin inside this outline was abraded using sterile fine-grit sand paper (previously autoclaved). The formerly prepared cell suspension was applied and gently rubbed on the abraded skin. Infected animals were randomized into nine groups (*n* = 5): a group of infected untreated animals, a group receiving miconazole only containing film (M), a group receiving miconazole/10% urea containing film (M + U10), a group receiving miconazole/20% urea containing film (M + U20), a group receiving miconazole/40% urea containing film (M + U40), a group receiving 10% urea containing film (U10), a group receiving 20% urea containing film (U20), a group receiving 40% urea containing film (U40), and a group receiving placebo film (P). Treatment by films started 72 h postchallenge and continued only daily for 7 days. The skin of guinea pigs should be wetted with sterile water using a sterile pipette tip before topical application of films (2.5 cm × 2.5 cm) to aid in its adhesion to the infected area. Infected untreated group did not receive the medicated films.

Clinical and mycological assessment of the treatment efficacy was performed. All groups were examined daily throughout the course of the experiment. Clinical evaluation was carried out first, then mycological assessment. Ten strands of hair were uprooted by sterile forceps inoculated on Sabouraud agar plates and incubated at 30°C for 2–4 days and examined by a stereomicroscope for mycological evaluation. Growth of the fungus on hair root was scored positive or negative. The number of infected hair roots was counted, and the effectiveness of the medicated film in decreasing the number of fungus-positive hair roots per treatment group was expressed as percentage relative to that of the infected untreated group using the following equation:

$$100-\left[T\times \left(\frac{100}{k}\right)\right]$$

where *T* is the total number of positive hair roots in the test group and *K* is the total number of positive hair roots in the infected untreated group.

Total score indicates the mean count of fungus-positive hair roots taken from animals in the same group.

For the clinical evaluation, local changes in the infected skin were given a score based on a scale from 0 to 5. No lesions were given 0, slight erythema 1, redness and swelling 2, rescaling and marked redness 3, partial integumental damage and hair loss 4, and extensive integumental damage and complete hair loss 5. The scores were summed for each animal and used to calculate the clinical efficacy of different treatments. It was expressed as a percentage relative to the result of the infected untreated group as follows:

$$100-\left[T\times \left(\frac{100}{k}\right)\right]$$

where *T* is the total number of positive hair roots in the test group and *K* is the total number of positive hair roots in the infected untreated group.

Total score indicates the mean clinical scores of animals in the same group (Garvey et al., 2015; Long et al., 2016).

#### Histopathology

Guinea pig infection and treatment were also evaluated by collecting dermal biopsy (5 mm in diameter) from anesthetized animals representing the group using punch on day 13. Specimens were rinsed with PBS. About 10% neutral buffered formalin was used for fixing tissues, which was then embedded in paraffin and processed for histopathological examination. Skin specimens were cut into 5-μm thick section and stained with H&E as well as Periodic acid–Schiff (PAS). The latter aid in the detection of fungal elements that appeared to be stained bright red (Saunte et al., 2008).

### Statistics

Independent repeating of experiments, at least three times, was performed to achieve reproducibility. Data display was in the form of mean and SD. Significant differences between groups were detected using one-way ANOVA, considering *P* < 0.05. Statistical analysis of data was done using IBM SPSS (17.0, IBM United States).

## Results

Using microdilution assay, the MIC of miconazole against *T. rubrum* was found to be 0.5 mg/L. A marked reduction in the MIC of miconazole was recorded when combined with 10% urea, 0.002 mg/L. In addition, increasing the urea concentration to 20% was associated with dramatic decrease in the MIC of miconazole to 0.001 mg/L. No further reduction in the miconazole MIC was obtained upon increasing the urea concentration to 40% as shown in Table 1. Moreover, the MFC of miconazole alone against *T. rubrum* was 1 mg/L. Interestingly, the MFC value was markedly reduced to 0.004 mg/L when the drug was combined with 10% urea. No further reduction in the MFC value of miconazole (0.004 mg/L) was recorded when combined with 20 or 40% urea. *T. mentagrophytes* showed similar findings as recorded in Table 1. Moreover, urea alone had no antimicrobial effect against the test fungi. These results indicated the presence of synergism between urea and miconazole against the test fungi, as the FIC values were below 0.5 as presented in Table 1. In addition, DMSO showed no effect on the fungal growth.

**TABLE 1:** Minimum inhibitory, minimum fungicidal concentrations and fractional inhibitory concentrations of miconazole against *T. rubrum* and *T. mentagrophytes* in the absence and presence of different concentrations of urea.

**Urea concentration (%)**	**MIC and MFC of miconazole**					
	**in (mg/L), and FIC against**					
	***T. rubrum***			***T. mentagrophytes***		
	**MIC**	**MFC**	**FIC**	**MIC**	**MFC**	**FIC**
0	0.5	1	–	1	2	–
10	0.002	0.004	0.004	0.002	0.004	0.002
20	0.001	0.004	0.002	0.001	0.004	0.001
40	0.001	0.004	0.002	0.001	0.004	0.001

**FIGURE 1:**
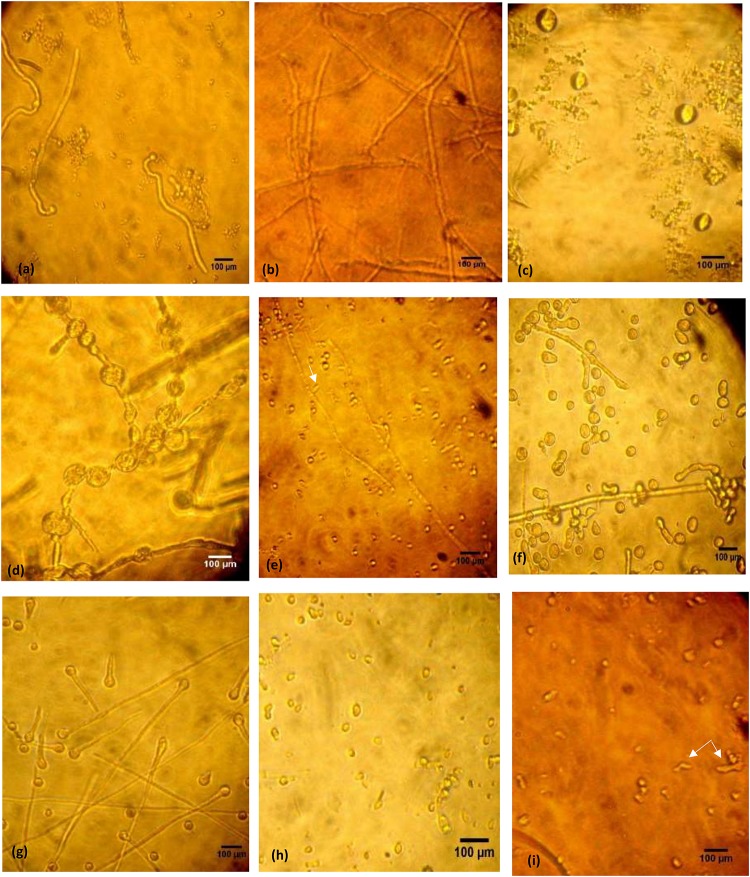
Microphotographs of control and treated *T. rubrum* grown in RPMI during 72 h at 30°C, using inverted phase-contrast microscope, 400× (bar 100 μm). Untreated fungus showing initiated germination 4 h following incubation of spores **(a)**. Normal forms of hyphae after 72 h **(b)**. Miconazole treated cells showing different modifications after 72 h; formation of conidia in 1 mg/L **(c)**, thick wider hyphae with vacuoles in 0.5 mg/L **(d)**, thin long hyphae with tear-shaped microconidia (arrow) in 0.25 mg/L **(e)**. Chlamydospore formation in 20% urea treated cells after 72 h **(f)**. Germination of spores in 10% urea treated cells **(g)**. Inhibition of spore germination by a combination of 20% urea and 0.004 mg/L miconazole detected after 72 h **(h)**. Deformities, cellular damages, and short necrotic hyphae (arrow) in 20% urea and 0.002 mg/L miconazole combination **(i)**.

Inverted microscopic examination revealed germination of the microconidia (Figure 1a). Furthermore, the untreated mycelium showed normal cell structures with homogeneous cytoplasm, no spores or chlamydospores, and long clear septate hyphae (Figure 1b). Morphological alterations were induced in *T. rubrum* following different treatments. For miconazole, the use of a fungicidal concentration (1 mg/L) resulted in the formation of swollen microconidia (Figure 1c). Shorter and wider hyphae with multiple vacuole-like structures inside them was observed following treatment with static concentration (0.5 mg/L), while treatment with a substatic concentration (0.25 mg/L) led to the production of thinner branched hyphae with lateral tear-shaped microconidia after 3 days (Figures 1d,e). The use of 0.004 mg/L miconazole did not show any changes compared to the control (data not shown). Upon subjecting the test fungus to 20% urea alone, chlamydospores were detected, while further decline in the concentration of urea (10%) led to spore germination, which was different than that of the untreated control, and this might be due to exposure to urea (Figures 1f,g). The use of fungicidal concentration of miconazole (0.004 mg/L) combined with 20% urea inhibited the germination of spores completely. However, treating the pathogen with subcidal concentration of miconazole (0.002 mg/L) combined with 20% urea led to a lot of deformities and severe damage in the fungal structures (Figures 1h,i). Similar effects were detected upon using fungicidal and subfungicidal concentrations of miconazole with either 10 or 40% urea (data not shown).

**FIGURE 2:**
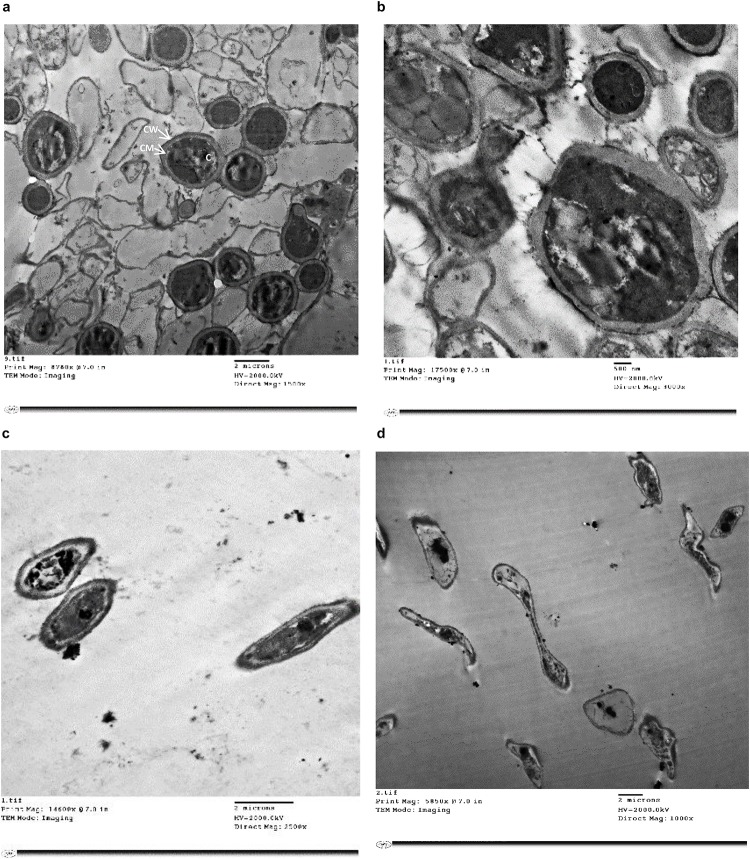
TEM photographs of treated and untreated *T. rubrum* showing **(a)** a normal morphology with intact cell walls, membranes and dense cytoplasm, **(b)** cells treated with 20% urea displaying slight cell wall irregularities and hazy edges but the cytoplasm remained dense, **(c)** cells treated with 1/2 MIC of miconazole showing less dense cytoplasm, **(d)** cells treated with a combination of 0.003 mg/L of miconazole/20% of urea showing degeneration. CW; cell wall, CM; cell membrane, C; cytoplasm.

**FIGURE 3:**
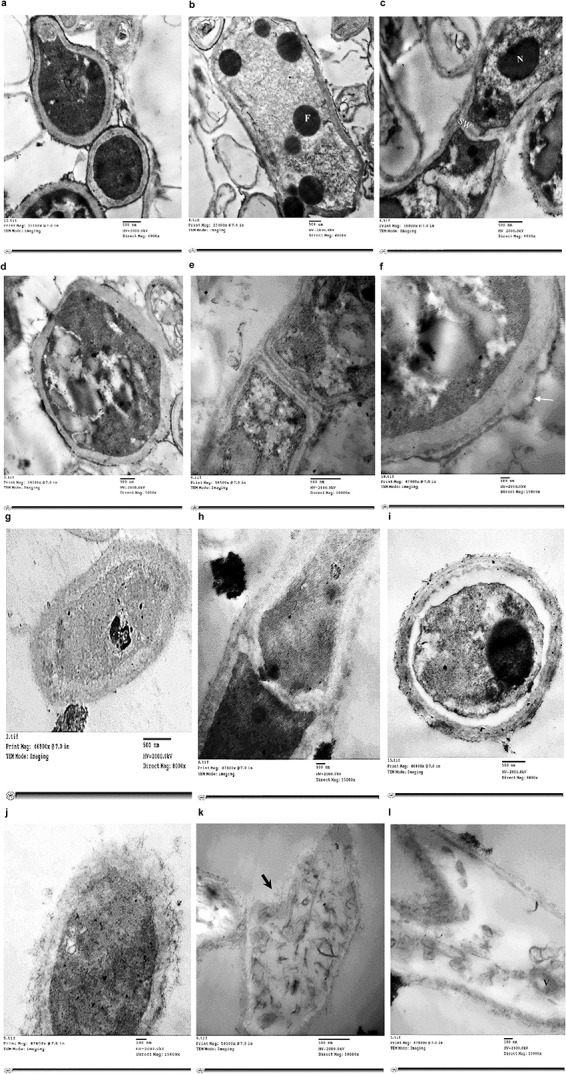
Electromicrographs of *T. rubrum* cells examined by TEM showing untreated cells with normal cell wall, membranes and cytoplasm **(a)**, some fatty droplets (F) appear as deep dark rounded spots **(b)**, normal septal walls (SW) of hyphae displaying lipid extruded via hyphal pore & nucleus (N) clearly distinguished **(c)**, some irregularities in urea -treated cells **(d)**, damage in the cell wall **(e)**, separated cell wall in urea-treated cells as pointed by the arrow **(f)**, less dense cytoplasm, thickened cell wall & normal hyphal septa in cells treated with 0.25 mg/L of miconazole **(g,h)**, plasmolysis & shrink cytoplasm with indented membrane in cells treated with 20% urea and 0.001 mg/L miconazole **(i)**, more thickened cell wall with severe damage as debris & disintegrated organelles in cells treated with 20% urea/0.002 mg/L miconazole **(j)**, necrotic cells with punctured wall (arrow, **k)**, collapsed cells with degraded cytoplasmic content & remnants of the membrane in the form of vesicles (V) within the cytoplasm in cells treated with 0.003 mg/L of miconazole combined with 20% urea **(l)**.

Treatment of *T. rubrum* by miconazole in the absence or presence of urea revealed different ultrastructures modifications, either at the periphery or inside the fungal cell, which were recorded using TEM. It showed a dense cytoplasm of the untreated cells as well as those treated with urea (Figures 2a,b). However, the cytoplasmic density was slightly reduced in miconazole-treated cells and dramatically decreased in *T. rubrum* cells subjected to 0.003 mg/L of miconazole/20% urea (Figures 2c,d). As shown in Figure 3a, a normal morphology, growth, and division of untreated *T. rubrum* cells were displayed. Intact cell wall, membranes, and dense cytoplasm were also detected. The presence of some saturated lipid droplets was observed. They appeared as deep dark rounded spots within the cytoplasm, as seen in Figure 3b. Normal septal walls of hyphae displaying the pores, plugs, and fats extruded via hyphal pore were observed (cytoplasmic translocation). In addition, the nucleus was clearly distinguished (Figure 3c).

Regarding 20% urea-treated cells, irregular cell wall was observed (Figure 3d). Moreover, septal wall of hyphal cells showed cytoplasmic translocation inhibition (Figure 3e). Damage and separation in the cell wall was also detected (Figures 3e,f).

For miconazole-treated cells (0.25 mg/L), less dense cytoplasm, thickened cell wall, and normal hyphal septa were detected (Figures 3g,h). Moreover, testing 0.004 mg/L of miconazole did not show any changes on the cellular ultrastructures compared to the control (data not shown).

Concerning the treatment of test fungal cells with a combination of miconazole and urea, the morphology as well as the ultrastructures had drastically changed showing the following: (i) plasmolysis and shrink cytoplasm with indented membrane in cells treated with 20% urea and 0.001 mg/L miconazole (Figure 3i); (ii) markedly thickened cell wall with severe damage appearing in the form of debris and disintegrated organelles in cells treated with 20% urea and 0.002 mg/L miconazole (Figure 3j); (iii) necrotic cells with punctured wall (Figure 3k); and (iv) collapsed cells with degraded cytoplasmic content and remnants of the membrane in the form of vesicles within the cytoplasm in cells treated with 20% urea and 0.003 mg/L miconazole (Figure 3l). Similar effects were also detected upon using substatic and subfungicidal concentrations of miconazole with either 10 or 40% urea (data not shown).

The composition of different water-soluble films is presented in Table 2. Formula 1 (F1) contained the basic film components, while F2–F6 represented the main film components with regular increasing amount of urea. F7–F11 contained different combinations of the penetration enhancer and the antifungal agent MFC. The physical examination of the films showed that all of them were transparent, smooth, uniform, and flexible.

**TABLE 2:** Composition of different formulae of water-soluble films.

**Formulae**	**Miconazol ug/cm^2^**	**Urea mg/cm^2^**	**CMC mg/cm^2^**	**PG mg/cm^2^**
Fl	0.00	0.00	9.26	0.03
F2	0.00	0.94	9.26	0.03
F3	0.00	0.189	9.26	0.03
F4	0.00	2.38	9.26	0.03
F5	0.00	3.77	9.26	0.03
F6	0.00	4.72	9.26	0.03
F7	0.19	0.94	9.26	0.03
F8	0.38	0.189	9.26	0.03
F9	0.57	2.38	9.26	0.03
F10	0.75	3.77	9.26	0.03
Fll	0.94	4.72	9.26	0.03

*F1–F11 representing different formulae composition, CMC is carboxymethyl cellulose, and PG is propylene glycol.*

Table 3 represents the tested physical characters of the plain film (F1), the amount of different urea concentrations (F2–F7), and that containing MFC of the active substance as well as the permeation enhancer (F7–F11). From Table 2, there was no significant difference between the ADC and the theoretical drug content (TDC). The thickness of the prepared films was slightly increased by increasing the concentration of urea, which was followed by a slight decrease upon addition of miconazole. At the same time, the thickness of drug-containing film was more than that of the plain film. SD values for all previously mentioned variables were low, indicating the reproducibility of the preparation method.

**TABLE 3:** Physicochemical properties of the prepared water-soluble films.

**Formulae**	**Theoretical weight (mg/4 cm^2^)**	**Weight (mg/4 cm^2^) (±SD)**	**Folding endurance**	**Thickness (mm)**	**pH**	**Theoretical drug content (±SD)**	**Drug content (mg/4 cm^2^) (±SD)**
F1	37.17	32 (1.82)	>200	0.220	6.82	0.000	0.000
F2	40.9	33 (1.51)	>200	0.250	6.74	0.000	0.000
F3	44.72	34 (1.85)	>200	0.255	6.72	0.000	0.000
F4	48.49	38 (1.67)	>200	0.260	6.72	0.000	0.000
F5	52.27	44 (1.79)	>200	0.260	6.69	0.000	0.000
F6	56.04	48 (1.69)	>200	0.260	6.68	0.000	0.000
F7	41.70	37 (0.99)	>200	0.255	6.43	0.76	0.69 (0.01)
F8	46.70	40 (1.79)	>200	0.240	6.45	1.510	1.44 (0.03)
F9	50.76	43 (1.43)	>200	0.245	6.48	2.265	1.12 (0.03)
F10	55.29	51 (1.33)	>200	0.240	6.52	3.020	2.81 (0.05)
F11	59.82	55 (1.89)	>200	0.240	6.54	3.774	3.30 (0.06)

The results of the folding and endurance are also recorded in Table 3. It was observed that films were capable to tolerate folding for more than 200 times without cracking. These results indicated the flexibility and the efficient elasticity of the prepared films, which is required for their application between fingers.

The palatability of the films is one of the general film tests, which was not recommended in this case because the prepared soluble film was intended to be applied between the fingers. The film palatability was normally controlled by measuring the surface pH values of the film. This was intended to assure the absence of dramatic pH changes in order not to affect pH of the skin following application of the medicated film. From Table 2, it can be noticed that there was absence of dramatic changes in the pH values of the prepared films, which ranged between 6.82 and 6.43.

One of the most important properties of the film was its ability to absorb water from the surrounding media, which aids in its solubilization and release of both drug as well as the permeation enhancer. An experimental model, to what in reality done, was developed and used for the first time. The results showed that the maximum absorption occurred after 10 min (for plain film) and 40 min (for drug-containing films); then, there was a decrease in the films’ weight. After 45 min, it was not possible to weight all films because all of them lost their film structure (Supplementary Figure 1).

**FIGURE 4:**
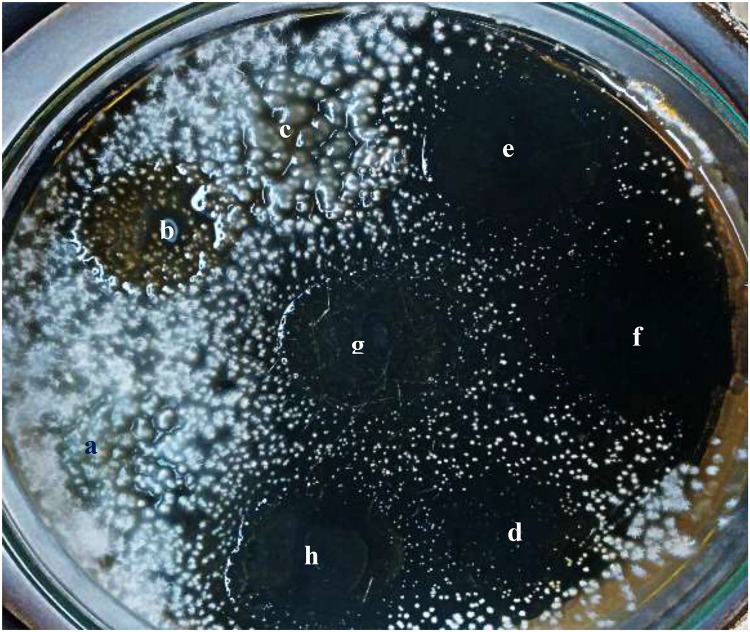
Antifungal activity of miconazole-containing film in the absence and presence of urea showing inhibited growth of *T. rubrum* around the films after 14 days of incubation at room temperature. **(a)** Placebo film, **(b)** miconazole containing film M, **(c)** 20% urea U20, **(d)** combination of miconazole and urea (M + U10), **(e,f)** combination of M + U20, **(g,h)** combination of M + U40.

Concerning the *in vitro* microbiological assessment of the antifungal activity of water-soluble films, they showed increase in the size (swollen) following 40 min of application on Sabouraud agar and incubation due to absorption of water from the culture medium (Supplementary Figures 2a–c). After 14 days of incubation at room temperature, there were no inhibition zones detected ∼0.004 mg/mL miconazole-, 10% urea-containing films, 20% urea, and 40% urea, against *T. rubrum*, and they were similar to the placebo film (Figures 4a–d and Table 4). However, films containing combinations of miconazole and increasing concentrations of urea displayed increase in the inhibition zone diameters. Interestingly, a significant (*P* = 0.037) increase in the diameters was achieved up to 40 mm when M + U20 was tested. The same result was also obtained by M + U40 combination as recorded in Table 4 and shown in Figures 4e–h. *T. mentagrophytes* showed similar results as recorded in Table 4.

**TABLE 4:** Assessment of *T. rubrum* and *T. mentagrophytes* growth inhibition by drug-containing water-soluble films using disc diffusion assay.

**Test organism**	Inhibition zone diameters in (mm)*							
	**U 10%**	**U 20%**	**U 40%**	**M**	**M + U 10%**	**M + U 20%**	**M + U 40%**	**Placebo film**
*T. rubrum* (ATCC 28188)	0 ± 0	0 ± 0	0 ± 0	0 ± 0	21 ± 0.06	44 ± 0.03	44 ± 0.05	0 ± 0
*mentagrophytes* (ATCC 24953)	0 ± 0	0 ± 0	0 ± 0	0 ± 0	33 ± 0.11	40 ± 0.07	40 ± 0.1	0 ± 0

*U, urea; M, miconazole (0.004 mg/L); each experiment was carried out in triplicates and the average was calculated ± standard deviation (SD). M, miconazole MFC.

**FIGURE 5:**
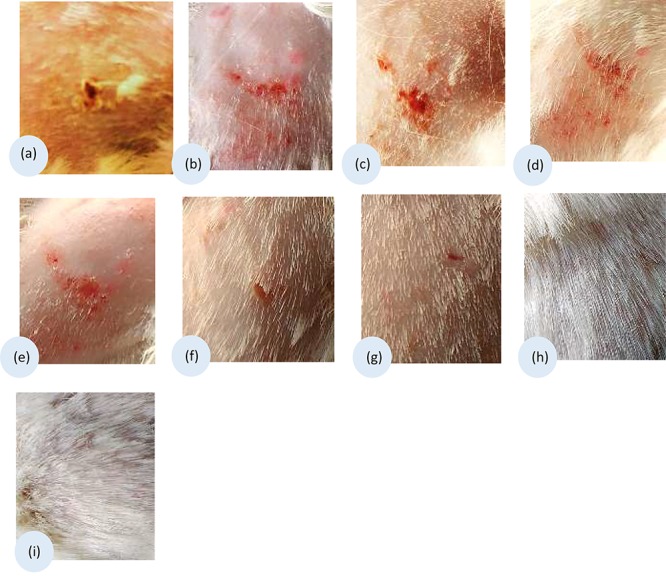
Clinical appearance of the infected sites of guinea pigs dermatophytosis model before, model group **(a)** and after subjection to different treatment using the medicated films, **(b)** placebo film (P); **(c)** U10; **(d)** U20; **(e)** U40; **(f)** Positive control treated with miconazole film (M); **(g)** combination treated group M + U10; **(h)** M + U20; and **(i)** M + U40. Representative photos of the infected areas captured on the 13th day of the study.

Regarding the clinical assessment, infected animals were monitored daily for the appearance of infection signs. By study day 4, guinea pigs showed redness of the infected area. The infected untreated control group showed scaly skin, patches of hair loss, and ulceration at the time of evaluation (study day 13) as shown in Figure 5a. Placebo, U10, U20, and U40 groups showed redness and ulceration (Figures 5b–e). Less improvement in the appearance of the animal skin was detected in case of treatment using miconazole alone (M) as well as miconazole/10% urea (M + U10) containing films but with a little hair growth (Figures 5f,g). In contrast, the treatment groups (miconazole/20% urea and miconazole/40% urea) showed normal hair growth with no signs of infection (Figures 5h,i).

Table 5 presents the clinical efficacy of the test films. The efficacy percentages for miconazole/urea combinations (M + U20 or M + U40) and placebo film were 95.83 and 0%, respectively, which resulted in high significant difference (*P* < 0.001). The percent efficacy for miconazole-containing film was 29.16%, which was significantly lower than that recorded for the above combinations (*P* < 0.001). Animals in miconazole/urea combinations (M + U10) group were non-significantly improved compared to the animals in the infected untreated control group, with a *P-*value of 0.06. Furthermore, the clinical efficacy of M + U20 was significantly better than that of M + U10 treatment (*P* < 0.001), but there was no difference between M + U20 and M + U40 combinations.

**TABLE 5:** Summary of mycological and clinical efficacies of different medicated water-soluble films.

	**Clinical efficacy**			**Mycological efficacy**		
**Water-soluble films**	**% efficacy**	**Mean clinical score ± SD**	***p*-value compared to untreated group**	**% efficacy**	**Mean clinical score ± SD**	***p*-value compared to untreated group**
M/U 40 film	95.83	0.2 ± 0.44^a^	0.001	95.83	0.2 ± 0.44^b^	<0.001
M/U 20 film	95.83	0.2 ± 0.44^c^	<0.001	95.83	0.2 ± 0.44^d^	<0.001
M/U 10 film	37.5	3 ± 0.71	0.06	33.3	3.2 ± 0.84	0.06
M	29.16	3.4 ± 0.45	0.08	25	3.6 ± 1.4	0.13
U40	8.35	4.4 ± 0.44	0.6	12.5	4.2 ± 0.83	0.51
U20	8.35	4.4 ± 0.44	0.3	4.16	4.6 ± 0.54	0.8
U10	0	4.8 ± 0.45	0.9	0	4.8 ± 0.45	0.35
Placebo films	0	4.8 ± 0.45	0.17	0	4.8 ± 0.45	0.64
Infected untreated group	–	4.8 ± 0.44	NA^e^	–	5 ± 0.0	NA

*^*a,b,c,d*^(*p*-value 0.001) to treatment and placebo film, ^*e*^NA not applicable.*

For mycological assessment, Table 5 shows also the mycological efficacy of the test films. The infected untreated group of animals recorded the highest average number of fungus-positive hairs. The efficacy percentages for miconazole/urea combinations (M + U20 or M + U40) and placebo solution were 95.83 and 0%, respectively. The percent efficacy for miconazole-containing film was 25%. Moreover, the mycological efficacy for miconazole/urea combinations (M + U20 and M + U40) groups was significantly better than that of the placebo as well as the infected untreated group (*P* < 0.001).

**FIGURE 6:**
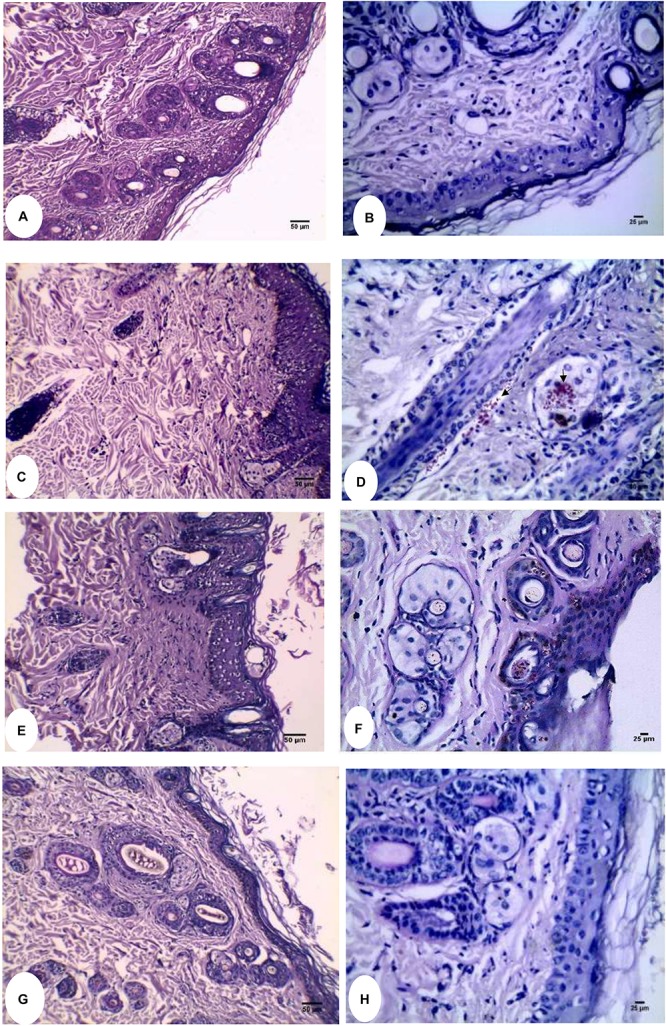
Histopathological evaluation of Guinea pig dermatophytosis model. **(A)** Normal uninfected control showing thin epidermis and normal dermal structure (H&E). **(B)** PAS-stained normal uninfected control free from the fungal elements. **(C)** H&E stained infected untreated control showing thick epidermis, hyperkeratosis, and distorted hair follicles. **(D)** Arthrospore and hyphal fragments appear bright red (PAS stained) within the hair follicle and sebaceous glands as pointed by arrows. **(E)** Miconazole received group showing little effect on the epidermal thickness of skin section stained with H&E. **(F)** Fungal elements were clearly detected in the epidermis as well as the hair follicle (PAS stained). **(G)** Miconazole/20% urea received group showing recovery of normal skin thickness and structure (H&E). **(H)** PAS stained skin section of miconazole/20% urea received group showing complete absence of fungal elements. At 200X **(A,C,E,G)** and 400X **(B,D,F,H)**.

Regarding the histopathological evaluation of H&E-stained skin sections, a normal skin structure was detected in Figure 6A, where normal epidermal and dermal structures and thickness were noticed. In addition, appropriate number of normal hair follicle, sebaceous glands, and sweat glands were observed in the dermis. For infected untreated skin section, increased epidermal thickness and hyperkeratosis were observable (Figure 6C). Moreover, there was decreased number of hair follicles with distorted shape where the root sheath cannot be detected. Infiltration of inflammatory cells into the dermal layer was also noticed. Failure of therapy using miconazole only was detected in Figure 6E where thick epidermis, hyperkeratosis, and infiltrated inflammatory cells were noticed. Interestingly, normal skin structure was recovered following treatment using the combination film (M + U20) as seen in Figure 6G. PAS-stained sections confirmed the above results where the fungal elements were observed entrapped within keratinous, epidermis, and some hair follicles in infected untreated group as well as the miconazole-treated group (Figures 6D–F). PAS negative sections see in Figures 6B,H. Groups of U10, U20, U40, and placebo showed results similar to that of the infected untreated control (data not shown). In addition, the findings of group M + U40 were the same like those of M + U20 (data not shown).

## Discussion

Recently, the incidence of dermatophyte infections has been increased considerably, particularly among elderly and pediatric populations as well as immunocompromised patients (Frías-De-León et al., 2020). Among these dermatophytes, *Trichphyton* spp. particularly *T. rubrum*, accounts for 69.5% of all dermatophytic infections and causes superficial dermatomycoses such as tinea pedis (Kemna and Elewski, 1996; Johnson, 2000). The clinical symptoms of tinea pedis involve moist white interdigital thick skin creating an optimum media for the fungi to multiply beneath the skin (Degreef, 2008).Then, the idea of our innovative pharmaceutical formula is to prepare a dosage form, which can absorb watery secretions from the infected area. This in turn helps to dry the infected skin and solubilize the film releasing the active substances. In addition, this pharmaceutical dosage form should have a bioadhesive character to remain in contact with the infected area. Furthermore, the presence of a penetration enhancer, like urea, will facilitate the drug diffusion through the thick skin. Therefore, the best pharmaceutical dosage form, which meets these criteria, is the water-soluble film with bioadhesive property.

Currently, there is a reasonable number of antifungal agents in the pharmaceutical market intended to control mycoses; however, tolerance or resistance to these drugs occurs due to their restricted cellular targets. For example, the subinhibitory concentrations of the antifungal agent promotes stress and leads to compensatory stress responses accompanied with upregulation of certain genes involved in drug efflux, cellular detoxification, and signaling pathways, and hence, these different mechanisms may contribute to tolerance (Martinez-Rossi et al., 2018). The use of drug combinations might result in a broader spectrum of activity and decrease the emergence of resistance against antifungal agents (Kingsbury et al., 2012). Within the context of screening for new drug combinations effective against *Trichophyton* spp., we evaluated whether urea had a synergistic inhibitory effect if combined with miconazole against *T. rubrum* and *T. mentagrophytes in vitro*. Our findings revealed that 10% urea combined with miconazole markedly decreased MIC and MFC to 0.002 and 0.004 mg/L, respectively, against the test fungi, indicating synergism (FIC < 0.5). Furthermore, increasing the concentration of urea to 20 or 40% in combination with miconazole reduced only MIC dramatically to 0.001, while it did not affect the MFC (0.004 mg/L). Mady et al. (2018) investigated the effect of miconazole/urea combination against resistant *C. albicans* and proved its effectivity in reducing MIC from 32 to 0.0625 μg/mL, resulting in a synergistic effect. Hultenby et al. (2014) reported that 50% topical K101 nail solution composed of urea, propylene glycol, and lactic acid was effective against *T. rubrum* cells.

Several reports documented the morphological and ultrastructural alterations of *T. rubrum* hyphal cells subjected to fungicidal and fungistatic concentrations of different antifungal agents. Hultenby et al. (2014) reported that *T. rubrum* cells treated with 50% topical K101 nail solution resulted in collapsed and degraded cells when examined by TEM. Pereira et al. (2011) and Song et al. (2018) reported that excessive production of chlamydospores, detected by phase contrast microscope, was stimulated by a high concentration of natural antifungal agents of plant origin. To our knowledge, these modified structures formed under unfavorable environmental conditions might be necessary for fungal survival in an adverse environment generated by antifungal agents. These cytological modifications might be attributed to the interference of the drugs with the enzymes responsible for cell wall synthesis (Song et al., 2018). Furthermore, Nishiyama et al. (1992) stated that amorolfine affected the enzymes responsible for the cell wall synthesis and caused deficiency in the ergosterol content. Nishiyama et al. (2017) reported that ME1111 exerted its antifungal activity via inhibition of succinate dehydrogenase and hence ATP-production discontinuation, as well as interference with active transport system present in the cell membrane with subsequent cell lysis. Moreover, cytoplasmic translocation inhibition was reported by Taplin and Blank (1961), who stated that the cytoplasm of higher fungi as *T. rubrum* is continuous through hyphae to enable filaments consisting of many cells to act as one unit in response to different stimuli. The plugging of the cytoplasm is a defense mechanism by which the fungus prevents loss of the cytoplasm into injured or dead neighboring cells. Interestingly, our results were in agreement with these findings.

Regarding preparation of water-soluble film, it was essential to add propylene glycol as a genuine plasticizer to the macromolecular chains of CMC to produce an elastic film. Propylene glycol is liquid in nature, and any added excess amount would not be entrapped within the film and remained as an unobservable thin layer following dryness of the film (Al-Badr, 1972). The excellent folding and endurance results of the prepared film might be due to using excess amount of propylene glycol. This effect might be attributed to two reasons: first, the amount of urea used was high compared to the film structure composition. Second, urea is a crystalline substance, which would be expected to increase the rigidity of the film (Al-Badr, 1972; Mady et al., 2018). To simulate the mechanism by which the inserted soluble film produce the antifungal effect, a special model was suggested. The local film solubility curve model represented the absorption of solution from the moist infected area, which was considered the first step in the treatment resulting in dryness of the infected area, to some extent, and release of the active substances to penetrate the deeper skin layers exerting a localized effect. The chemical interaction between the drug and the film component could be expected as reported by Mady et al. (2018). They proved the disappearance of all characteristic drug peaks under those of the film components following application of IR scan.

Disk diffusion method was used to evaluate the antifungal activity of drug-containing films against *T. rubrum* and *T. mentagrophytes in vitro*. This method is known to be reliable, practical, and reproducible (Mahboubi and Kazempour, 2015). In this method, the test drug diffused from the film through a solidified Sabouraud agar layer to an extent inhibited the growth of the added dermatophyte, forming a zone around the films containing effective concentrations of miconazole alone or in combination with urea. In our study, the diameters of inhibition zones reached up to 40 mm when 20% urea was combined with 0.004 mg/L miconazole, which is a very low concentration of miconazole. The latter result was also obtained upon testing miconazole with 40% urea, indicating that the use of 20% urea in combination with MFC of miconazole was enough to get effective *in vitro* inhibitory effect against *Trichophyton* spp. Gupta and Kar (2015) tested a nanoformulation of miconazole against *T. rubrum* that showed inhibition zone diameters up to 14 mm at 250 mg/L, while the plain drug displayed 10 mm, indicating that our preparation was more effective.

Regarding guinea pig dermatophytosis model, the clinical and mycological efficacies of the test films were also assessed. The combinations of M + U40 and M + U20 showed the same and the highest efficacy percentage (95.83%), which was statistically superior to the infected untreated control (*P* < 0.001) in fungal burden reduction as well as improvement in the clinical scores (*P* < 0.001). This effect indicates that the use of 20% urea was enough as a penetration enhancer with miconazole for treatment of fungal infection and no need to increase the urea concentration above 20%. Similar findings were reported by Garvey et al. (2015) who stated that VT-1161 compound improved the onychomycosis clinically as well as mycologically, supporting the clinical development of VT-1161 as an antifungal agent. Zhou et al. (2016) mentioned that analysis of skin sections of guinea pig infected untreated group revealed the presence of fungal elements, occasional inflammation, tissue destruction, and acanthosis that were clearly observed in PAS as well as H&E-stained sections. On the other hand, complete absence of fungal elements and tissue recovery were observed in the treatment group using CK100% formulation.

## Conclusion

Given the clinical importance of skin fungi and the need to expand antimicrobial options, our study evaluated the antimicrobial activity of the miconazole/urea combination against *T. rubrum* and *T. mentagrophytes* due to the absence of reports in the literature on their combined effect on conidia and fungal mycelial growth. Synergistic interaction was recorded for such compounds *in vitro* as well as *in vivo*. In addition, this combination was associated with clinical and mycological improvement in the guinea pig dermatophytosis model when applied topically as water-soluble medicated film, providing a possibility of combination therapies that potentiate the spectrum of antifungal activity while diminishing the chance of drug resistance. Furthermore, this innovative pharmaceutical dosage form is now involved in a clinical trial on patients in Tanta University Hospital.

## Data Availability Statement

All datasets generated for this study are included in the article/Supplementary Material.

## Ethics Statement

The animal infection protocol was reviewed and approved by Animal Care and Use Committee at the Faculty of Pharmacy, Tanta University, Tanta, Egypt with approval number (REC-TP/M0003).

## Author Contributions

LA-M and OM conceived the experiments and analyzed the results. LA-M, AD, and OM conducted the experiments. All authors wrote and reviewed the manuscript.

## Conflict of Interest

The authors declare that the research was conducted in the absence of any commercial or financial relationships that could be construed as a potential conflict of interest.
