# Supplementary material for: Preparation, and Assessment of Antidermatophyte Activity of Miconazole–Urea Water-Soluble Film
Source: Front Microbiol. 2020 Apr 3;11:385. doi: 10.3389/fmicb.2020.00385 (PMC7145891; doi:10.3389/fmicb.2020.00385)
Supplement: Supplementary file 1 [file Data_Sheet_1.pdf]

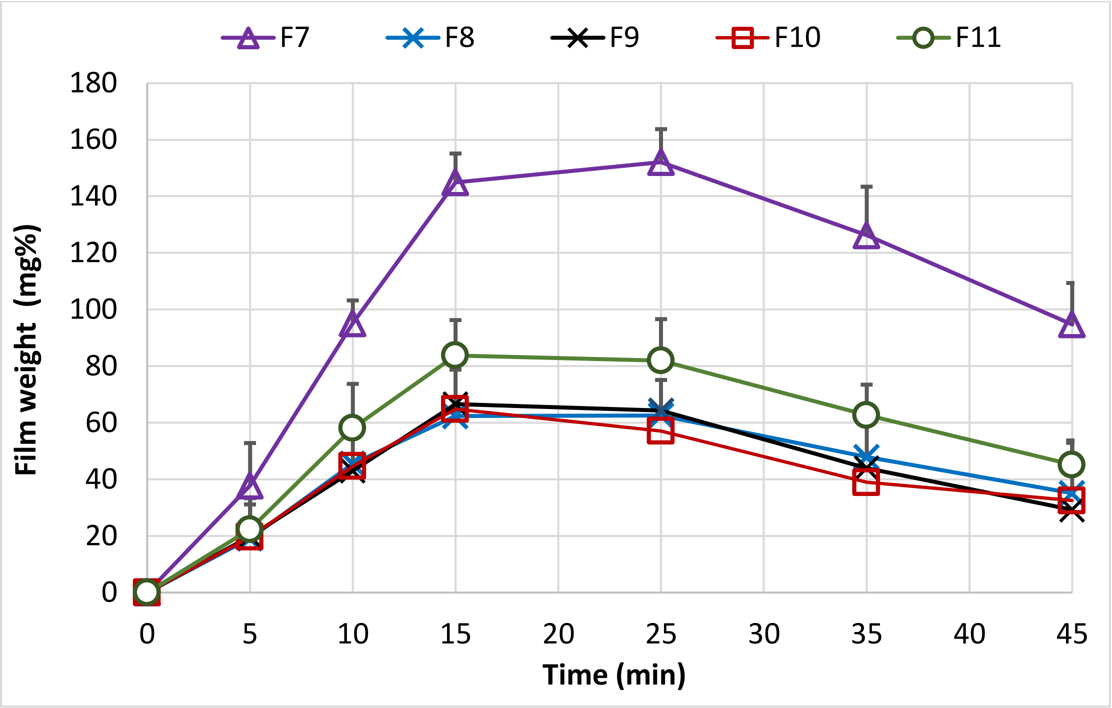

Figure (S1): Swelling and solubility of the different prepared medicated films in the suggested local film solubility model. The bars represent the standard deviation.

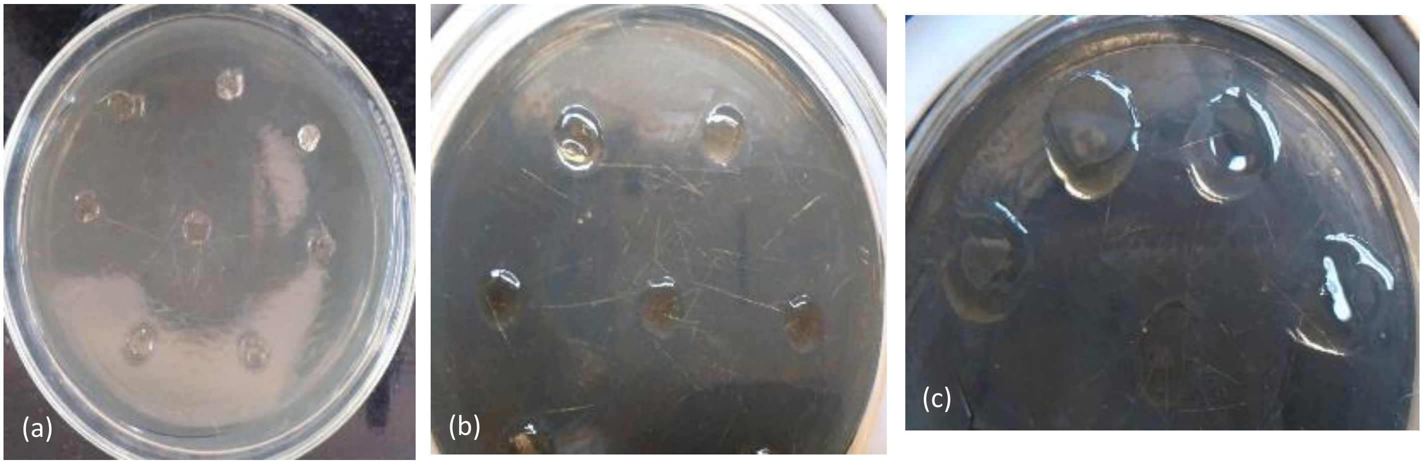

Zero time after 10 min After 40min of incubation

Figure (S2): Application of water-soluble films on sabouraud agar seeded with T. rubrum showing swelling of the film due to absorption of water from the medium after 10 min of incubation at room temperature (b), with continuous increase observed after 40 min of incubation (c) compared to those at zero time of incubation (a).
